# Hospital Construction

**Published:** 1897-12-18

**Authors:** 


### HOSPITAL CONSTRUCTION.

### BERBICE COUNTY HOSPITAL, NEW AMSTERDAM.

Berbice Hospital is of modern construction and in its way is a model of a tropical hospital. The building is handsome and has a really imposing entrance. It is fortunate in having a nice pleasure ground and shubbery, rich in flowers. Like all buildings in these regions it is well raised on pillars from the ground to avoid the malaria, as the whole country is rich mud flat, and lies low. Operating-room, male ward, and several small paying patients' wards are here, and everything spotlessly clean. The floors, polished now, used to be scrubbed. A small bench, beautifully clean, between the beds serves as a table. The basins and all utensils were arranged on these. There are several small private wards for paying patients. They are very simply furnished with wire mattresses and mosquito curtains, a couple of chairs and small table, and washstand. Screens are placed between the beds so that each patient has a certain amount of privacy. A long table is provided for bottles, with antiseptics, washes, and liniments. The wards are airy and looked exceedingly clean. Between each bed a white wooden table is fixed, with a rim and rounded corners. Most of these were very clean, so were all bowls and utensils. This is the more noticeable as it was the only institution in the country where I could honestly say things smelt sweet and fresh. The head male nurse is a coloured man, and was neat and clean, deft in ward work. His two assistants appeared intelligent. In the middle of the wards basins, &c., were arranged on tables. The head nurses' table was covered with a bright coloured cloth, while gay coloured blankets over the beds gave a cheery appearance. A number of coolies were patients, most of them with malarial fever or slight accidents, cuts and bruises, ulcers, &c. A great many of them could not speak even the broken English prevalent in the colony. One bright-eyed boy was very anxious to show his bad foot, which was poisoned, but doing well.

On each floor, on either side of the stairs, was a small, compact scullery for each ward, with cup and bowls neatly arranged, and washing-up was being done by a bright-eyed negress.

The women's wards were all in good order, and managed by two nurses under the matron, a comfortable-looking Dutchwoman, but who was getting beyond her duties. She did not live on the premises, but was on duty 12 hours. The matron's room and linen-room was bare-looking; adjoining it is a very pleasant convalescent ward, but used at present for patients' cases. A good view of the town and river can be seen from the tower. The kitchen stands a little way from the main building, and was orderly and clean, boilers and saucepans being beautifully kept.

Under a shed in the grounds old mattresses were being picked and re-made; cocoa-nut fibre is used for this. Great credit is due to the medical officer, who takes great pains, and is doing a great deal to make nurses thoughtful and careful, and anxious to do as well as their English sisters. Dr. Ross, the surgeon-general, is very anxious to introduce all female nurses, but this must be a work of time. Most of the medical men say the class of men that offer for nursing is inferior to the females. One of the female nurses was not pleasant to look at, one side of her face and shoulder being drawn and distorted; she had entered as a patient two years ago, and was kept on as nurse, more from pity than anything else. Thoroughly trained, conscientious nurses would indeed be a great boon. There is the great question of extra expense; times are not good, and there is a most deadly apathy over everything.

